# Epidemiology, laboratory diagnosis and clinical aspects of fungal pulmonary infections in 384 patients hospitalized in pulmonary units in Guilan province, Iran

**DOI:** 10.18502/ijm.v12i4.3940

**Published:** 2020-08

**Authors:** Zahra Rafat, Seyed Jamal Hashemi, Keyhan Ashrafi, Iraj Nikokar, Alireza Jafari, Abbas Rahimi Foroushani, Behrad Roohi, Zeinab Borjian Boroujeni, Niki Najar-Shahri

**Affiliations:** 1 Department of Medical Parasitology and Mycology, School of Public Health, Tehran University of Medical Sciences, Tehran, Iran; 2 Food Microbiology Research Center, Tehran University of Medical Sciences, Tehran, Iran; 3 Department of Medical Microbiology, School of Medicine, Guilan University of Medical Sciences, Rasht, Iran; 4 Medical Biotechnology Research Center, School of Paramedicine, Guilan University of Medical Sciences, Rasht, Iran; 5 Urology Research Center, Razi Hospital, Guilan University of Medical Sciences, Rasht, Iran; 6 Department of Statistics and Epidemiology, School of Public Health, Tehran University of Medical Sciences, Tehran, Iran; 7 Department of Medical Mycology, Mazandaran University of Medical Sciences, Sari, Iran

**Keywords:** Bronchoalveolar lavage, Sputum, Candidiasis, Fungal respiratory infections, Invasive pulmonary aspergillosis, Galactomannan antigen

## Abstract

**Background and Objectives:**

The respiratory tract is the most common site for developing fungal infections. People who have a weakened immune system are more susceptible to respiratory system involvement with fungi. Fungal infections of the respiratory tract are largely unrecognized and their true burden is elusive. Therefore, the aim of the current study was to evaluate the clinical spectrum, demographic characteristics, risk factors, and etiology of fungal respiratory infections in 384 patients hospitalized in pulmonary units of Razi hospital, Guilan province, Iran.

**Materials and Methods:**

A total of 384 lung specimens (192 Bronchoalveolar lavages (BAL) and 192 sputa) were obtained from patients who met the inclusion criteria. All samples were analyzed by direct microscopy and culture. Fungal identification was accomplished by internal transcribed spacer (ITS) and beta-tubulin sequencing. Also, in patients suspected to invasive pulmonary aspergillosis BAL specimens were tested for galactomannan (GM) antigen. According to the host factors (clinical symptoms, radiology findings and predisposing factors which were defined as inclusion criteria), and the positive results in direct examination, culture and serology (GM for aspergillosis) the infection was confirmed.

**Results:**

Fungal respiratory infection was confirmed in 137 cases (35.67%) including 86 (62.77%) males and 51 (37.23%) females and the highest prevalence of infection was found in the age group of 46–72 years (n=75, 54.74%). Cough (n=129, 94.16%), dyspnea (n=111, 81.02%), purulent sputum (n=85, 62.04%) and weight loss (n=77, 56.2%) were the predominant symptoms. Tuberculosis (n=34, 24.81%), taking chemotherapy regimen (n=30, 21.89%) and diabetes mellitus (n=27, 19.70%) were the predominant underlying conditions. *Candida albicans* (37.22%) and *Candida tropicalis* (21.89%) represent the two most commonly isolated species in the current study. Furthermore, according to revised EORTC/MSG (2008) definitions for invasive fungal infections, from 5 cases of pulmonary aspergillosis, 2 (40%) cases of probable invasive pulmonary aspergillosis (IPA) and 3 (60%) cases of possible IPA were diagnosed.

**Conclusion:**

According to the results of this study, infected infants with congenital CMV infection could identify at early stage by testing Guthrie cards (within 21 days of life). Furthermore, since there is a lack of CMV knowledge in our population, educating and effective counseling by obstetricians/gynecologists to the pregnant women are recommended.

## INTRODUCTION

Respiratory tract infections are globally responsible for one-third of infectious disease-associated mortality, accounting for 4.3 million annual deaths. Despite treatment, most invasive pulmonary fungal infections are associated with high mortality rates of > 50% (1, 2). The air we breathe is filled with thousands of fungal spores (conidia). After inhalation these tiny elements, hosts may have no symptoms or may cough up blood or have a fever or chest pain or may have symptoms ranging from allergies to life-threatening invasive mycoses (3, 4). The outcome depends on the immune status of the host. During recent decades, fungal respiratory infections are being recognized with increasing frequency in the world in parallel with an expanding population of immunocompromised patients, such as those with human immunodeficiency virus (HIV), cancer and transplant patients who are taking certain immunosuppressive drugs (5).

In Iran, the prevalence of candidiasis has doubled in some hospitals, especially in intensive care units. In most Iranian reports in the past two decades, *Aspergillus* has been the second most common cause of invasive fungal infections that have been isolated from patients (6).

In the last decade, less well-known molds, such as members of the *Fusarium* and *Scedosporium* genera, and newly described *Aspergillus* species are increasingly found to cause invasive pulmonary infection. Also, *Cryptococcus gattii* has emerged in North America as a cause of pulmonary infection (7).

The incidence and etiology of pulmonary fungal infections can vary in various types of patient’s hospital settings, and geographical locations. In one study, *Aspergillus* spp. were isolated from 33% (86/251 cases) of lung-transplantation recipients, which involved colonization (n=50), tracheobronchial lesions (n=17) or invasive aspergillosis (n=19) (8). Also, invasive pulmonary aspergillosis mortality of neutropenic patients was 40 to 60% in early reports (9). *Candida* infection was reported as the most dominant pulmonary fungal infection in patients with non-hematologic malignant tumors and in non-lung solid organ transplant recipients. The diagnosis of this serious infection is so problematic and it can annually cause many deaths around the world.

Taken together, the clinicians must remain vigilant for invasive and serious fungal lung infections even to individuals who were considered only moderately immune-compromised. The purpose of the current study was to determine the clinical spectrum and etiology of fungal respiratory infections and identify the underlying risk factors in 384 hospitalized patients in Razi hospital, Rasht, Iran.

## MATERIALS AND METHODS

### Ethics statement.

Informed consent to take part in the study has been obtained from subjects or their guardians prior to sample collection. This study was approved by ethical committee of Tehran University of Medical Sciences (the number of Ethics Committee protocol: IR.TUMS.SPH.REC.1397.002).

### Sampling.

This descriptive cross-sectional study was carried out over a period of two years, from October 2017 to October 2019 in Razi hospital, the lung care center of Guilan province, Iran. The presence of two or more following conditions was used as inclusion criteria in this study:

(1) Patients who displayed at least one of the following host factors: receiving chemotherapy within the last 3 months before admission in order to treat solid tumors, chronic obstructive pulmonary disease (COPD), steroid use: at least 4 mg methylprednisolone (or equivalent) per day for at least 7 days in the past 3 weeks before admission or a cumulative dose of at least 250 mg of methylprednisolone (or equivalent) in the past 3 months before enrollment and recipient of any other immunosuppressive treatment (tacrolimus, cyclosporine, methotrexate, cyclophosphamide, and sirolimus).
(2) Patient with clinical symptoms indicative of pulmonary fungal diseases according to a pulmonary diseases specialist opinion (dyspnea, cough, high and persistent fever, recurrent fever, chest pain, purulent sputum, weight loss, night fever, hemoptysis, rhinitis, and wheezing)
(3) Patients with suspicious radiographic findings indicative of pulmonary fungal diseases according to a pulmonologist opinion.

Patients who had taken any systemic antifungal agents before enrollment for treating infections other than pulmonary fungal disease were excluded from the study in order to prevention of false-negative results.

A total of 384 lung specimens including 192 Bronchoalveolar lavage (BAL) and 192 sputum samples were obtained from patients hospitalized in pulmonary units of Razi hospital. Demographic features including age, gender, underling disease and patient’s clinical manifestations (fever, purulent sputum, dyspnea, etc.) were recorded. Samples were collected in sterile dry containers and transported to laboratory. Sputum samples were concentrated and BAL samples were centrifuged and the deposit was used for examination. For direct microscopic examination, the samples were dissolved in KOH 10% solution and observed under a microscope for fungal elements. Calcofluor white staining (Sigma, Deisenhofen, Germany) was done to detect mycelial and yeast cells and Indian ink was used to check for *Cryptococcus neoformans* (10, 11). All specimens were cultured on Sabouraud Dextrose Agar (SDA) with chloramphenicol and Brain Heart Infusion (BHI) agar media (Merck, Germany). Culture media were surveyed after incubation at 30 and 37°C for 48–72 hours. Culture media for those cases with no growth were maintained up to two weeks. Any growth obtained was further identified by its rate of growth, colony morphology and lactophenol cotton blue mounts. Slide culture was performed as required (12, 13). Yeast isolates were identified based on production chlamydoconidia in cornmeal agar (Becton, France) and colony color on chromogenic CHROMagar *Candida* medium (CHROMagar, Paris, France) (14). Also, for all patients with a positive direct examination of the BAL sample in addition to isolation of *Aspergillus* species in culture who were suspected to invasive pulmonary aspergillosis (IPA) BAL specimens were tested for galactomannan (GM) antigen with ELISA technique. Furthermore, for confirmation of diagnosis, all isolates were subjected to PCR and sequencing techniques.

### BAL GM test.

Platelia Aspergillus GM EIA (Bio-Rad, France) was used to measure the galactomannan of lavage samples according to the manufacturer procedures (15). Briefly, 300 μl of serum or BAL fluid was added to 100 μl of treatment solution, boiled for three minutes at 104°C and then centrifuged for 10 minutes in 10000× g. Next, 50 μl of supernatant and 50 μl of conjugate were mixed and incubated in microtiter plates precoated with monoclonal antibody EB-A2 for 90 minutes at 37°C. The plates were washed five times; after which they were incubated with 200 μl of tetramethylbenzadine in the dark for 30 minutes. The reaction was stopped by 100 μl of sulfuric acid and absorbance at 450 and 620 nm read using a plate reader. Positive and negative controls were included in each assay. Results were recorded as an index relative to the optical density (OD) of the cut-off control. The GM of lavage and serum was considered positive when OD index was ≥ 0.5. All positive cases were repeated in the same sample before they were considered positive.

### Molecular technique.

DNA Extraction: Fungal genomic DNA was extracted from harvested colonies using glass bead disruption method (16).

### PCR conditions and sequencing.

PCR amplification for each isolate was performed as described previously (17, 18). To discriminate *Aspergillus* isolates at the species level the Beta tubulin gene of *Aspergillus* species was amplified using the forward (Bt2a: 5′-GGTAACCAAATCGGTGCTGCTTTC-3′) and reverse (Bt2b: 5-ACCCTCAGTGTAGTGACCCTTGGC-3) primers. Also, for identification of other fungal isolates in the species level the universal primers used for fungal amplification were ITS1 (5′TCC GTA GGT GAA CCT GCG G 3′), which hybridizes at the end of 18S rDNA, and ITS4 (5′TCC TCC GCT TAT TGA TAT GC 3), which hybridizes at the beginning of 28S rDNA (Life Technologies, Barcelona, Spain).

Positive PCR products were sent for sequencing at Bioneer Advanced Nucleic Acids core facility. Sequences were then parsed from the coting and separately used to perform individual nucleotide–nucleotide searches using the BLASTn algorithm at the NCBI website (https://blast.ncbi.nlm.nih.gov/Blast.cgi).

Fungal identifications were made based on maximum identities ≥ 99% and query coverage ≥ 98% with this method. All sequences were deposited in Gen-Bank under the accession number reported in Table 1.

**Table 1.** GenBank accession numbers of DNA sequences included in this study.

**Fungal elements**	**GenBank accession numbers used in the sequence analysis**
*Candida albicans*	MK793223, MN419311, MN394878, MK138363, MH729024, KX355315, MG020722, KP674532, GQ280312, KC905076, HE860439, JN606216, AB369945, MK113223, JN606273, JN60625, AB018037, AB018038, LC522889, LC522889, MH016316, KY101869, KY101873, KY101874, KY101875, KY101901, KY101906, MG913256, KY996543, MF614746, MF614741, MF614725, MF614723, JN882314, JN882321, GQ376070, EF192231, AF455428, MN318604, MH729028, KC905069, MG818819, MG818824, KY996544, KY996539, MH016327, MH016296, MH734813, MH729024, MG599201, MN4193373, MN419366, MH918807, MK307750, GQ280305, GQ849400, EF568101, JX406290, KU095860, AB436996
*Candida tropicalis*	MK793225, MT028124, MN919090, MN796064, MG009522, MK547223, HM231275, GQ376071, EU924133, AY939810, MH534930, MH534908, MG720231, KU950724, GQ376071, EU924133, KU987879, KM361510, MH545915, MK394119, MH591472, MH260384, KX198669, KJ451708, KJ451647, KF746430, KF746416, EU288196, KJ451642, KC254014
*Candida glabrata*	FN652301, GQ376080, AM492798, AM492797, MK793223, KU99239, KU992391, KU992392, KU992393, LC317498, FN652301, LS398111, LC311497, LR757911, KP131708, LR757911, FN652302, HE993756, LS398123, LC317498, FN652301, AY939793
*Candida krusei*	MH545928, FJ515204, JX174414, MK394162, LC389008, KF959838, MH545928, MN310532, MK894151
*Candida parapsilosis*	MK394127, KY102205, KP131738, LN864530, MG241512, LC317527, DQ681358, EU564209
*Rhodotorula mucilaginosa*	KT876599, MN913572, KT87670, KP960513, MN427959
*Trichosporon asahii*	MN809474, AB018013, AB018014
*Geotrichum candidum*	MH443758, KX928847, MK499446
*Aspergillus flavus*	MK119732, AY017536, M38265
*Rhizopus oryzae*	LC514310
*Aspergillus niger*	LC387867
*Aspergillus fumigatus*	MH781327
*Alternaria alternata*	MK793206

### Statistical tests.

The data analysis was performed by SPSS software (V.20). The study was assessed by using standard Chi-squared and 95% Confidence intervals (CI). Statistically, P value < 0.5 was considered as significant difference or correlation.

## RESULTS

Totally 384 hospitalized patients (including 238 Males and 146 females) suspected to fungal respiratory infections according to inclusion criteria were enrolled. Of this population, in 137 patients (35.67%), respiratory fungal infection was proved. The patients were within the age range of 1–86 years and the highest prevalence of respiratory fungal infections was found in the age group of 46–72 years (n=75, 54.74%) and showed the age of subjects was not significantly effective on the incidence of fungal respiratory infections (P=0.546) (Table 2).

**Table 2.** The distribution of 137 patients with pulmonary fungal infections hospitalized in pulmonary units based on age groups, gender and underlying diseases

			**Patients**				**P-value^a^**

			**Number**		**Percentage**		
Age groups (years)	1–45		33		24.08		0.546
	46–72		75		54.74		
	73–86		29		21.16		
Gender	Female		86		62.77		0.8111
	Male		51		37.23		
Underlying diseases	Tuberculosis		34		24.81		<0.001
		Breast cancer		9		6.56	
		Prostate cancer		7		5.1	
	Solid tumors	Ovarian cancer	30	6	21.86	4.37	
		Lung cancer		4		2.92	
		Esophageal adenocarcinoma		3		2.1	
		Osteosarcoma		1		0.72	
	Diabetes mellitus		27		19.70		
	Kidney failure		10		7.29		
	Heart failure and hypertension		10		7.29		
	Respiratory failure		10		7.29		
		Rheumatoid arthritis		3		2.18	
	Autoimmune diseases	Multiple Sclerosis	8	2	5.83	1.46	
		Systemic lupus erythematosus		2		1.46	
		Pemphigus vulgaris		1		0.72	
	Without underlying disease		4		2.92		
	Iron deficiency anemia		2		1.46		
	AIDS ^b^		1		0.72		
	Asthma		1		0.72		
Total			137		100		

a P-value, probability value;

b AIDS, Acquired immunodeficiency syndrome common symptoms.

The ratio of infected females to males was 51 (37.23%) to 86 (62.77%) and statistical analysis could not find any association between the distribution of hospitalized patients with proven respiratory fungal infections and patient’s gender (P= 0.811) (Table 2).

Previous history of tuberculosis, taking chemotherapy regimens at the time of study (in order to treat solid tumors) and diabetes mellitus were found in 24.81%, 21.86% and 19.70% of patients, respectively. These predisposing factors were significantly associated with the occurrence of respiratory system involvement with fungi (P=0.000) (Table 2).

**Fig. 1.**
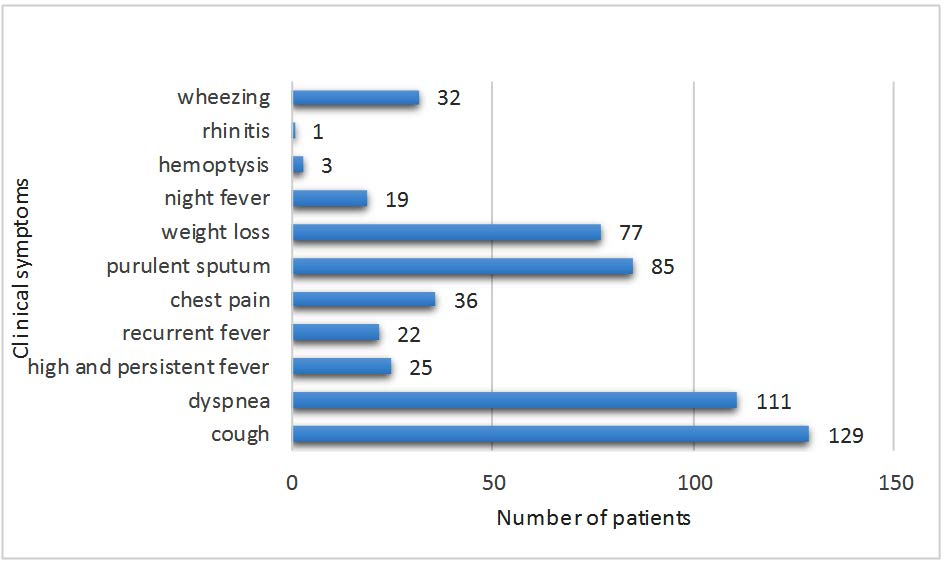
The clinical spectrum and different symptoms among 137 hospitalized patients with respiratory fungal infections

Among 137 positive cases, cough (n=129, 94.16%), dyspnea (n=111, 81.02%), purulent sputum (n=85, 62.04%) and weight loss (n=77, 56.2%) were the most there was a significant relationship between developing respiratory fungal infections and clinical symptoms (P=0.000) (Fig. 1).

Statistical analysis showed that *Candida albicans* (37.22%) and *Candida tropicalis* (21.89%) represent the two most commonly isolated species in the current study followed by *Candida glabrata* (12.4%), *Candida krusei* (5.83%), *Candida parapsilosis* (5.1%), *Trichosporon asahii* (2.18%), *Geothricum candidum* (2.18%), *Aspergillus flavus* (2.18%), *Rhizopus orizae* (0.72%), *Aspergillus niger* (0.72), *Aspergillus fumigatus* (0.72%) and *Alternaria alternata* (0.72%). Also, mixed fungal elements in some examined specimens were detected in this study (Table 3).

**Table 3.** The frequency distribution of fungal elements isolated from 137 patients with pulmonary fungal infections hospitalized in pulmonary units

**Isolated fungi**	**Frequency**	**Percent**
*Candida albicans*	51	37.22
*Candida tropicalis*	30	21.89
*Candida glabrata*	17	12.4
*Candida krusei*	8	5.83
*Candida parapsilosis*	7	5.1
*Candida glabrata* + *Candida albicans*	5	3.56
*Rhodotorula mucilaginosa* + *Candida albicans*	4	2.92
*Trichosporon asahii*	3	2.18
*Geotrichum candidum*	3	2.18
*Aspergillus flavus*	3	2.18
*Rhizopus oryzae*	1	0.72
*Aspergillus niger*	1	0.72
*Aspergillus fumigatus*	1	072
*Alternaria alternata*	1	0.72
*Candida krusi* + *Candida parapsilosis*	1	0.72
*Rhodotorula mucilaginosa* + *Trichosporon asahii*	1	0.72
Total	137	100

In this study, criteria such as presence of invasive forms (numerous budding yeasts, pseudohyphae and true hyphae) in direct examination or significant growth of pure creamy mucoid colonies on culture media were considered to distinguish between *Candida* colonization and infection of the respiratory tract in patients who met the inclusion criteria (1, 19). According to these defined criteria from 137 patients with respiratory fungal infections, in 124 cases (90.51%) respiratory candidiasis was reported. In 46 cases (37.1%) forming pseudohyphae in direct smear and in 33 cases (26.61%) forming pseudohyphae in direct smear along with significant growth of pure creamy mucoid colonies on culture media were observed in mycological tests of patients with respiratory tract candidiasis (Table 4).

**Table 4.** Frequency distribution of respiratory tract candidiasis in 137 hospitalized patients with respiratory fungal infections base on defined criteria for *Candida* respiratory infection in direct examination and culture.

**Criteria for *Candida* respiratory infection in direct examination and culture**	**Frequency**	**Percent**
Forming pseudohyphae in direct smear	46	37.1
Significant growth of pure creamy mucoid colonies on culture media	29	23.39
Forming numerous budding yeasts in direct smear	12	9.68
Forming pseudohyphae in direct smear + significant growth of pure creamy mucoid colonies on culture media	33	26.61
Forming numerous budding yeasts in direct smear + significant growth of pure creamy mucoid colonies on culture media	4	3.22
Total	124	100

From 8 patients suspected to pulmonary aspergillosis, 5 cases (62.5%) of IPA were reported. All of them had positive results in direct examination with forming branching septate hyphae along with positive results in culture media. In 3 patients (2.19%) *Aspergillus flavus* was the etiologic agent of invasive aspergillosis, in one patient (0.72%) *A. fumigatus* was the etiologic agent and in one other patient (0.72%) *A. niger* was the cause of respiratory infection. *A. flavus* was the most common cause of invasive aspergillosis in present study.

Also during the period of this study GM tests were performed for all patients suspected to invasive aspergillosis. In this EIA test the OD index of 0.5 or more was taken as positive result (Table 5).

**Table 5.** GM cut-off value in BAL related to 8 patients suspected to invasive pulmonary aspergillosis hospitalized pulmonary units based on gender

**Gender** **GM ^a^ cut-off value** **In BAL ^b^ specimens**	**Women**		**Men**		**Total**	
		
	**number**	**%**	**number**	**%**	**number**	**%**
<0.5	2	25	2	25	4	50
≥0.5	1	12.5	3	37.5	4	50
Total	3	37.5	5	62.5	8	100

a GM, galactomannan;

b BAL, Bronchoalveolar lavage

It should be noted that in the current study, all sequencing results were consistent with macroscopic and microscopic identifications of the isolates.

According to revised EORTC/MSG (2008) definitions for invasive fungal infections (20), from 5 cases of pulmonary aspergillosis, 2 (40%) cases of probable IA and 3 (60%) cases of possible IA were diagnosed in this study (Table 6).

**Table 6.** Detailed information of each patient involved with invasive pulmonary aspergillosis categorized based on EORTC/MSC ^a^ (2008) criteria

**Row**	**Age (years)**	**Gender**	**Underlying disease**	**Symptoms**	**GM ^c^ level in BAL ^d^ specimen**	**CT ^e^ scan**	**Direct examination**	**Culture**	**IPA^f^**
1	30	Male	AIDS^b^	Cough, dyspnea, weight loss	0.7	-	Branching septate hyphae	*A. fumigatus*	Possible
2	53	Male	Solid tumor	Recurrent fever, chest pain, cough	2.3	Nodular lesions	Branching septate hyphae	*A. flavus*	Probable
3	35	Female	Lupus	Cough, dyspnea, Purulent sputum	0.5	-	Branching septate hyphae	*A .flavus*	Possible
4	63	Female	Solid tumor	Cough, Purulent sputum, dyspnea	0.2	-	Branching septate hyphae	*A. niger*	Possible
5	71	Male	Solid tumor	Hemoptysis, dyspnea, chest pain	2.1	Cavity	Branching septate hyphae	*A. flavus*	Probable

a EORTC/MSC; European Organization for Research and Treatment of Cancer and Mycoses Study Group;

b AIDS, Acquired immunodeficiency syndrome;

c GM, galactomannan;

d BAL, Bronchoalveolar lavages;

e CT scan, computerized tomography scan;

f IPA, Invasive pulmonary aspergillosis

Also in this study, from 137 hospitalized patients with proven respiratory fungal infections, one case of mucormycosis (0.72%) in a 76 years old man with prostate cancer was reported. After culture on SC medium and sequencing, *Rhizopus oryzae* was reported as the etiologic agent. Also one case of *Alternaria alternata* (0.72%) was isolated from the BAL sample belonged to a30 years old woman with a history of chronic nasal congestion and drainage, asthma and allergic rhinitis from 8 years ago. Also, 3 cases (2.19%) of geotrichosis due to *Geotrichum candidum* and 4 cases (2.92%) of trichosporonosis due to *Trichosporon asahii*, were reported in this study.

## DISCUSSION

In the course of their lives humans often come into contact with fungi that are present in the biosphere and the lungs are organs especially predisposed for fungal infections. Most fungal infections occur after inhalation of ubiquitous mycelial fungi in the environment. Also some yeast and yeast-like organisms are opportunistic fungal agents found as part of the normal microflora in human respiratory tract. Respiratory tract infections are globally responsible for one-third of infectious disease–associated mortality, accounting for 4.3 million annual deaths (2, 21). Despite treatment, most invasive fungal infections are associated with high mortality rates of > 50% (2, 22). In this study we evaluated the spectrum of fungal organisms causing infection in 384 patients hospitalized in pulmonary units and in 137 (35.67%) cases respiratory fungal infection was confirmed. In agreement with our results Basiri Jahromi et al. reported the same prevalence for fungal respiratory infections in Iran (23). Also Roohani AH et al. reported a higher prevalence of 26.7% for respiratory fungal infections in immunocompromised patients in India (24). While the Egyptian study of Ahmed and colleagues showed that the prevalence of fungal pneumonias in respiratory intensive care units was 66.67% (25). This inconsistency may be a result of the different patterns of age grouping, diversity in study designs and the different inclusion criteria for patients defined by each research group.

Most of patients in current study (54.74 %) were at an age range of 46–72 years old. Older adults become more susceptible to infections due to predisposing factors such as diabetes, renal insufficiency and arthritis. Also, when people age, there is immunosenescence, which means that the immune system doesn’t function as well or as vigorously. The combination of predisposing factors and the decrease in activity of the immune system can make these age group more prone to infections (26).

In this work, in accordance with the results of another report (23) the ratio of infected females to males was 51 (37.23%) to 86 (62.77%) and there was no significant difference in the prevalence of fungal respiratory infection between the genders. Similar exposure to pollution sources due to equal socio-demographic condition, occupations and responsibilities for men and women in society could be the reason for the similarity observes in the incidence of fungal respiratory infection between two gender groups. Cough (n=129, 94.16%), dyspnea (n=111, 81.02%), purulent sputum (n= 85, 62.04%) and weight loss (n=77, 56.2%) were the most common symptoms among 137 patients with fungal respiratory infection. Considering the fact that fungal respiratory infections often cause symptoms that are similar to other illnesses, such as the flu or tuberculosis, clinical and laboratory findings should be used simultaneously for making the final decision on drug administration.

Tuberculosis was the main predisposing factor observed in 24.81% of patient in current study. Tuberculosis (TB) is still one of the biggest killers among the infectious diseases especially in developing countries like Iran (27). This country shares geographic borders with three countries in which, tuberculosis is endemic: Afghanistan, Iraq and Pakistan. In addition, Iran is in a close association with other countries where tuberculosis is highly prevalent, i.e. China, India, Nepal, Bosnia, Bangladesh, Tajikistan, Sri Lanka and Azerbaijan. It should be noted that the percentage of mycotic infections increase in pulmonary tuberculosis patients. The results of different studies showed that physicians should pay particular attention to fungal co-infection with pulmonary TB (28). A history of taking chemotherapy regimens (21.89%) and diabetes mellitus (19.70%) were the other common underlying conditions for developing fungal respiratory infections. Studies showed that after intensive chemotherapy, the estimated risk of developing invasive pulmonary fungal infection is about 5%, and the reported mortality ranges from 30 to 80% (29). On the other hand, diabetic patients are very often prone to fungal infections, because of higher blood glucose levels which help for the growth of fungi. Diabetics have an immune system with a lower ability to respond to and deal with infections of any type. This means they are more prone to illnesses than the general population. Diabetes can contribute to the development of fungal/bacterial/viral pneumonia, tuberculosis and chronic obstructive pulmonary disease (COPD) (30).

During the laboratory analysis of specimens, a set of 165 fungal isolates including 152 (92.12%) yeasts and 13 (7.88%) molds were recovered from 137 patients. *Candioda albicans* (n=62, 37.57%) and *Candida tropicalis* (n=33, 20%) were the most common isolated species in this study. In Accordance with our results, Spahr J et al. reported that *Candida albicans* and *Candida tropicalis* are the most important causes of pulmonary involvement (31). *Candida* species are normal microflora of mucosal surfaces like respiratory tract. In this anatomical site, there is a balance between normal fungal flora and normal bacterial flora. When this balance is disturbed, *Candida* colonization is replaced by infection due to the creation of invasive forms (forming budding yeast cells, pseudohyphae and true hyphae in the specimen or by significant growth in the form of pure creamy mucoid colonies on culture media (1).

Also in current study, from 5 patients with IPA, in 3 patients (60%) *Aspergillus flavus* was the etiologic agent of invasive aspergillosis. Since in the most of studies, *Candida albicans* and *Aspergillus flavus* were specified as the most prevalent fungal etiology of respiratory infections (31–34), uncommon species like *Geothricum candidum, Trichosporon asahii, mucorales* fungi and non-*albicans Candida* species should not be ignored. Given that some uncommon species are intrinsically resistant to routine antifungal drugs, they could cause treatment failure and should be taken into account.

Also according to revised EORTC/MSG (2008) definitions for invasive fungal infections, from 5 cases of invasive pulmonary aspergillosis, 2 (40%) cases of probable IPA and 3 (60%) cases of possible IPA were diagnosed in this study. Studied showed that with increased use of immunosuppressive agents in recent years, the incidence rates of IPA have increased up to 30% in some centers (35–38). A BAL or serum enzyme-linked immunosorbent assay (ELISA) can help us for early diagnosis of IA with galactomannan (GM) detecting, which is a major constituent of *Aspergillus* cell walls. Raoul Herbrecht et al. in an assay evaluated over a 4-year reported a large-scale evaluation of *Aspergillus* GM ELISA (39). They stated that of the 67 probable cases of IA, 19 cases were defined by a single positive sputum or tracheal aspiration culture. Also in a large study of GM ELISA performed in allo-HSCT patients, 19 (76%) of the 25 patients that had proven aspergillosis and 14 (93%) of the 15 patients that had probable aspergillosis had positive antigenemia tests (40). To the best of our knowledge, this is the first study on the epidemiology, clinical spectrum, underlying conditions, and demographic characteristics associated with pulmonary fungal infections in Guilan province, located in Iran’s northern region.

## CONCLUSION

The prevalence of pulmonary fungal infections in Guilan province was 35.67%. Tuberculosis, receiving chemotherapy within the last 3 months before admission in order to treat solid tumors and diabetes mellitus were important risk factors, and *Candida albicans* was the most common fungal species responsible for pulmonary fungal infection. The highest prevalence of pulmonary fungal infections was found in the age group of 46–72 years and in male patients. Many physicians missed fungal pulmonary infection because it does not show specific clinical manifestations. Given that some of uncommon causal agents of fungal pulmonary infections are intrinsically resistant to routine antifungal drugs and could cause treatment failure, mycological examinations should be considered for proper treatment.
